# Association study of urinary iodine concentrations and coronary artery disease among adults in the USA: National Health and Nutrition Examination Survey 2003–2018

**DOI:** 10.1017/S0007114523001277

**Published:** 2023-12-28

**Authors:** Zhijian Wu, Meng Li, Jiandi Liu, Feng Xie, Yang Chen, Shuai Yang, Xiaozhong Li, Yanqing Wu

**Affiliations:** Department of Cardiology, The Second Affiliated Hospital of Nanchang University, 330006 Nanchang, Jiangxi, People’s Republic of China

**Keywords:** Urinary iodine concentration, Coronary artery disease, J-shaped, Diabetes

## Abstract

Iodine is a vital trace element in the human body and is associated with several important coronary artery disease (CAD) risk factors. We aimed to explore the correlation between urinary iodine concentration (UIC) and CAD. Data from 15 793 US adults in the National Health and Nutrition Examination Survey (2003**–**2018) were analysed. We conducted multivariable logistic regression models and fitted smoothing curves to study the correlation between UIC and CAD. Furthermore, we performed subgroup analysis to investigate possible effect modifiers between them. We found a J-shaped association between UIC and CAD, with an inflection point at Lg UIC = 2·65 μg/l. This result indicated a neutral association (OR 0·89; 95 % CI 0·68, 1·16) between UIC and CAD as Lg UIC < 2·65 μg/l, but the per natural Lg [UIC] increment was OR 2·29; 95 % CI 1·53, 3·43 as Lg UIC ≥ 2·65 μg/l. An interaction between diabetes and UIC might exist. The increase in UIC results in an increase in CAD prevalence (OR 1·84, 95 % CI 1·32, 2·58) in diabetes but results in little to no difference in non-diabetes (OR 0·98, 95 % CI 0·77, 1·25). The J-shaped correlation between UIC and CAD and the interaction between diabetes and UIC should be confirmed in a prospective study with a series of UIC measurements. If excessive iodine precedes CAD, then this new finding could guide clinical practice and prevent iodine deficiency from being overcorrected.

Iodine is one of the crucial trace elements for humans. It is generally understood that two-thirds of the iodine is in the thyroid gland, but organs other than the thyroid, such as the heart, kidneys and liver, are found to have iodine^(1)^. Although iodine is essential for human growth, development and function, inappropriate levels of iodine have harmful effects on the human body. Overwhelming evidence shows that both iodine deficiency and iodine excess can result in thyroid disorders such as hyperthyroidism, hypothyroidism, goitre, thyroid cancer and even an increased risk of miscarriage and infant death^(2–5)^.

Research has proven that iodine plays an important role in thyroid hormone synthesis, which influences cardiovascular function and metabolism (e.g. cardiac contractility, vascular tone, blood pressure (BP) and lipid metabolism)^(6,7)^, making iodine a critical factor in maintaining cardiovascular health. However, most studies on iodine have focused on the thyroid gland, and its effect on CVD has not yet been fully investigated. Coronary artery disease (CAD) is a CVD that is recognised as the major cause of mortality worldwide^(8)^, manifesting itself as stable or unstable angina, myocardial infarction or sudden cardiac death^(9)^. Current evidence suggests that iodine is associated with several important risk factors for CAD, such as BP, blood glucose and lipid metabolism^(10–14)^. However, the independent relationship between iodine and CAD has rarely been studied. Based on the above facts, we hypothesise that there is a strong correlation between iodine and CAD, which, if proven in the future by a large sample size of prospective trials, would have significant implications for public health. Moreover, to gain insight into the mechanisms underlying the association between iodine and CAD and to help identify high-risk groups that may benefit from targeted interventions, we propose to conduct a subgroup analysis of common high-risk factors for CAD (e.g. hypertension, diabetes, age, sex, BMI, smoking, etc.) to examine the effect of iodine status on CAD risk.

Urinary iodine concentration (UIC) analysis is the most recommended and commonly used biochemical method for evaluating iodine status in populations due to its ability to directly reflect body iodine status^(15,16)^. In this study, we extracted data from the representative National Health and Nutrition Examination Survey (NHANES) from 2003 to 2018 to investigate the correlation between UIC and CAD among adults in the USA and to explore the potential modifiers of the relationship between UIC and CAD.

## Methods

### Data source and population

NHANES is a nationally representative survey of the United States general population using a complex, multi-stage, probability sample design that gathers demographic, socio-economic, dietary and health-related data about the American public in a two-year cycle (www.cdc.gov/nchs/nhanes/index.htm). In this study, we planned to investigate the relationship between UIC and CAD among American adults by extracting data from eight cycles of NHANES (2003–2018). The National Center for Health Statistics ethics review board supported the NHANES research plan. All participants provided written informed consent. There were 80 312 participants in the NHANES from 2003 to 2018. After the exclusion of participants less than 18 years old (*n* 32 549), participants with missing CAD history data (*n* 2975), urinary iodine data (*n* 27 361) and participants with cancer (*n* 1634), the data from 15 793 participants were included in our analysis (online Supplementary Fig. 1).

### Coronary artery disease

Akin to the previously conducted NHANES research, in the NHANES questionnaire, participants were asked if they had ever been told by a doctor or other health professional that they had angina, CAD or myocardial infarction. If participants said ‘yes’ to the above questions, they were identified as having CHD^(17,18)^. Questionnaires are administered to NHANES participants both at home and in mobile examination centres. Selected persons are invited to take part in the survey by being interviewed. Interviews are conducted by highly trained health professionals, and each question is standardised.

### Urinary iodine and other measurements

There was no fasting or special diet prior to urine collection. Professionally trained researchers used sterile collectors with multiple urine screens to obtain adequate urine samples (optimal 1·8 ml, minimum 1·0 ml). The total urine iodine concentration was measured by inductively coupled plasma kinetic reaction cell mass spectrometry at the Laboratory Sciences of the National Center for Environmental Health, Radiation Analytical Toxicology Division. The UIC was divided into two groups according to the inflection point. Furthermore, we also collected the following potential covariates: demographic records including age, sex, race, marital status, education levels and poverty income ratio; body measurements including weight, height, BMI (the calculation method is weight divided by height squared) and waist circumference; disease and personal history, including smokers, drinkers, hypertension, thyroid dysfunction and diabetes. Standard biochemical tests include HbA1c, fasting plasma glucose (FPG), total cholesterol, TAG, HDL-cholesterol, serum creatinine, uric acid and the estimated glomerular filtration rate (eGFR, we calculated by the modification of diet in renal disease equation). Three BP readings were obtained after 5 min of participants resting quietly with their feet on the floor and back propped up. Based on these recorded readings, the average systolic and diastolic BP was calculated, that is, the participants’ BP. Thyroid dysfunction was defined as self-reported thyroid dysfunction, and hypertension was defined as self-reported hypertension or systolic BP ≥ 140 mmHg and/or diastolic BP ≥ 90 mmHg^(19)^. Diabetes was described as a self-reported diagnosis by a physician or FPG ≥ 7·0 mmol/l or HbA1c ≥ 6·5 %^(20)^.

### Statistical analysis

UIC was a skewed distribution in our study. Therefore, we conducted Log10 transition (Lg UIC) in the data analysis. The mean values and standard deviations or median (Q1, Q3) are used to describe continuous data, and numbers (%) to describe categorical data, respectively. In comparing differences in data characteristics with and without CAD, Student’s *t* test was used for normally distributed data, the nonparametric Mann-Whitney test for abnormally distributed data and the *χ* ^2^ test for categorical data. Confounders were included in the final models if they were correlated with the CAD or an effect change estimate of more than 10 %^(21)^. Online Supplementary Tables S1–S4 show the associations of each confounder with CAD. Multivariable logistic regression analysis evaluated the correlation between UIC (continuous and categorical variables) and CAD. We conducted three regression analysis models: model 1 was unadjusted. Model 2 was adjusted for sex, age and race. In model 3, in addition to the adjustment in model 2, education levels, marital status, poverty income ratio, BMI, waist circumference, hypertension, diabetes, thyroid dysfunction, smokers, drinkers, FPG, total cholesterol, TAG, uric acid, serum creatinine, eGFR, HbA1c and HDL-cholesterol were considered. In addition, given that NHANES uses a complex, multi-stage sampling design, and therefore each individual’s contribution to the population is biased. To eliminate this bias, we also weighted sample data using weights to provide a final unbiased and accurate estimate of effects for the population.

As far as we know, many factors in biomedical research do not have a simple linear relationship with the outcome variable; most independent variables have no effect or a positive effect on the outcome variable within a certain range, beyond which the magnitude or/and direction of the effect changes. To clearly reflect the dose–response relationship and threshold effects between UIC and CAD, we first use a generalised additive model and smooth curve fitting to examine whether the UIC is partitioned into intervals. We apply segmented regression (also known as piece-wise regression) that is using a separate line segment to fit each interval. Log-likelihood ratio test comparing a one-line (non-segmented) model to segmented regression model was used to determine whether threshold exists. The inflection point that connecting the segments was based on the model gives maximum likelihood, and it was determined using two steps recursive method.

Analysis of interactions and stratification was performed based on age (< 60 or ≥ 60, years), sex (male or female), race (Mexican American, other Hispanic, non-Hispanic White, non-Hispanic black, or other race), BMI (< 25, ≥ 25 and < 30, ≥ 30, kg/m^2^), eGFR (< 60 or ≥ 60, ml/min/1·73 m^2^), hypertension (yes or no), diabetes (yes or no), thyroid dysfunction (yes or no), smokers (yes or no), drinkers (yes, no, or unknown) and LDL-cholesterol (known or unknown). Statistical analyses were performed on the R Pack (http://www.R-project.org) and EmpowerStats (http://www.Empowerstats.Com, X&Y Solutions, Inc.). A two-sided *P* < 0·05 was considered statistically significant.

For the numerous missing data, dummy variables were used to indicate missing covariate values, and sensitivity analyses were conducted to evaluate differences in demographic and clinical characteristics, the UIC and the incidence of CAD. Specifically, 55·39 % (*n* 8748) of the data were missing for LDL-cholesterol. For the minor missing data, we used multiple imputations, based on five replications and a chained equation approach method in the R MI procedure, to account for missing data. Specifically, 8·66 % (*n* 1368) of the data were missing for the poverty income ratio, 0·98 % (*n* 154) were missing for weight, 0·97 % (*n* 151) were missing for height, 1·11 % (*n* 175) were missing for BMI, 4·32 % (*n* 683) were missing for waist circumference, 5·04 % (*n* 796) were missing for FPG, 3·82 % (*n* 603) were missing for HbA1C, 4·77 % (*n* 754) were missing for total cholesterol, 5·10 % (*n* 805) were missing for TAG, 13·97 % (*n* 2206) were missing for HDL, 5·08 % (*n* 801) were missing for uric acid and 5·05 % (*n* 797) were missing for Scr and eGFR.

## Results

### Baseline participant characteristics

As shown in Table 1, the data from 15 793 participants were statistically analysed; 48·04 % were male, and 975 (6·17 %) had CAD. Among participants with CAD, the mean values and standard deviation for age and Lg UIC were 65·42 (sd 12·59) and 2·27 (sd 0·50), respectively. The CAD and non-CAD groups were statistically differentiated in baseline characteristics, except for the height and proportion of LDL-cholesterol missed. CAD individuals were likely to be older, male, White, have hypertension, diabetes, thyroid dysfunction, be smokers and drinkers; have lower values for education level, poverty income ratio, HDL-cholesterol and eGFR; and have higher levels of weight, BMI, waist circumference, FPG, HbA1c, uric acid, serum creatinine and UIC than those without CAD. Participants’ weighted baseline characteristics were largely similar compared with previous results, except in terms of race and educational attainment. In the weighted results, the proportion of non-Hispanic Whites is greater, and the educational attainment is higher, which is consistent with the national context of the USA (online Supplementary Table S5).

**Table 1.** Baseline characteristics of study participants (Mean values and standard deviations; numbers and percentages)

Variables*	Non-CAD (*n* 14 818)		CAD (*n* 975)		
	*n*	%	*n*	%	*P*
Age, years					
Mean	46·91		65·42		< 0·001
s d	17·09		12·59		
Male	7119	48·04	600	61·54	< 0·001
Race					< 0·001
Mexican American	2664	17·98	112	11·49	
Other Hispanic	1477	9·97	91	9·33	
Non-Hispanic White	5908	39·87	532	54·56	
Non-Hispanic Black	3237	21·85	178	18·26	
Other race	1532	10·34	62	6·36	
Marital status					< 0·001
No	2878	19·42	69	7·08	
Yes	11 940	80·58	906	92·92	
Education level					< 0·001
< 9th grade	1720	11·61	186	19·08	
9–11th grade	2209	14·91	163	16·72	
High school	3441	23·22	262	26·87	
College	4233	28·57	229	23·49	
Graduate or above	3215	21·70	135	13·85	
Poverty income ratio					
Median	2·12		1·62		< 0·001
Q1–Q3	1·11–4·09		0·95–2·92		
Weight, kg					
Mean	81·29		83·97		< 0·001
sd	21·35		21·46		
Height, cm					
Mean	167·16		167·12		0·915
sd	10·17		9·83		
BMI, kg/m^2^					
Mean	29·01		30·00		< 0·001
sd	6·84		6·92		
Waist circumference, cm					
Mean	98·51		105·07		< 0·001
sd	16·16		15·58		
Hypertension					< 0·001
No	9340	63·03	244	25·03	
Yes	5478	36·97	731	74·97	
Diabetes					< 0·001
No	12 834	86·61	621	63·69	
Yes	1984	13·39	354	36·31	
Thyroid dysfunction					< 0·001
No	13 517	91·22	821	84·21	
Yes	1301	8·78	154	15·79	
Smokers					< 0·001
No	8359	56·41	375	38·46	
Yes	6459	43·59	600	61·54	
Drinkers					< 0·001
No	3924	26·48	225	23·08	
Yes	696	4·70	96	9·85	
Unknown	10 198	68·82	654	67·08	
LDL-cholesterol					0·787
Know	8212	55·42	536	54·97	
Unknown	6606	44·58	439	45·03	
	Mean	sd	Mean	sd	
FPG, mmol/l	5·61	2·11	6·51	2·78	< 0·001
HBA1c, %	5·70	1·04	6·24	1·33	< 0·001
TC, mmol/l	5·07	1·08	4·69	1·18	< 0·001
TAG, mmol/l					
Median	1·37		1·55		0·008
Q1–Q3	0·90–2·10		1·06–2·32		
HDL-cholesterol, mmol/l	1·37	0·42	1·27	0·40	< 0·001
UA, umol/l	321·60	84·63	354·65	91·41	< 0·001
SCR, umol/l	77·39	30·92	95·85	44·75	< 0·001
eGFR, ml/min/1·73 m^2^	95·76	26·96	76·09	25·50	< 0·001
UIC, μg/l					
Median	137·20		171		< 0·001
Q1–Q3	78·20–239·50		93·05–298·60		
Lg UIC†, μg/l	2·14	0·39	2·27	0·50	< 0·001

FPG, fasting plasma glucose; TC, total cholesterol; UA, uric acid; SCR, serum creatinine; eGFR, estimated glomerular filtration rate; UIC, urinary iodine concentration.

* Data are presented as mean ± standard deviation or median (Q1–Q3) and numbers (%) as appropriate.

† UIC value was log10-transformed.

### The relationships between urinary iodine concentration and coronary artery disease

**Fig. 1.**
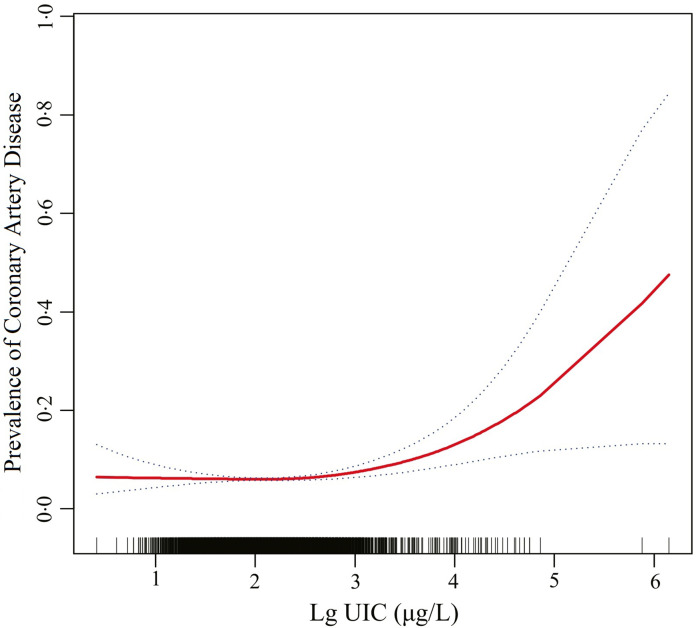
Association between urinary iodine concentration and the prevalence of coronary artery disease. The solid red and blue dotted lines represent the estimated values and their corresponding 95 % CI. Adjustment factors included sex, age, race, education levels, marital status, poverty income ratio, BMI, waist circumference, hypertension, thyroid dysfunction, diabetes, smokers, drinkers, fasting plasma glucose, total cholesterol, TAG, uric acid, serum creatinine, estimated glomerular filtration rate, HbA1c and HDL-cholesterol.

**Fig. 2.**
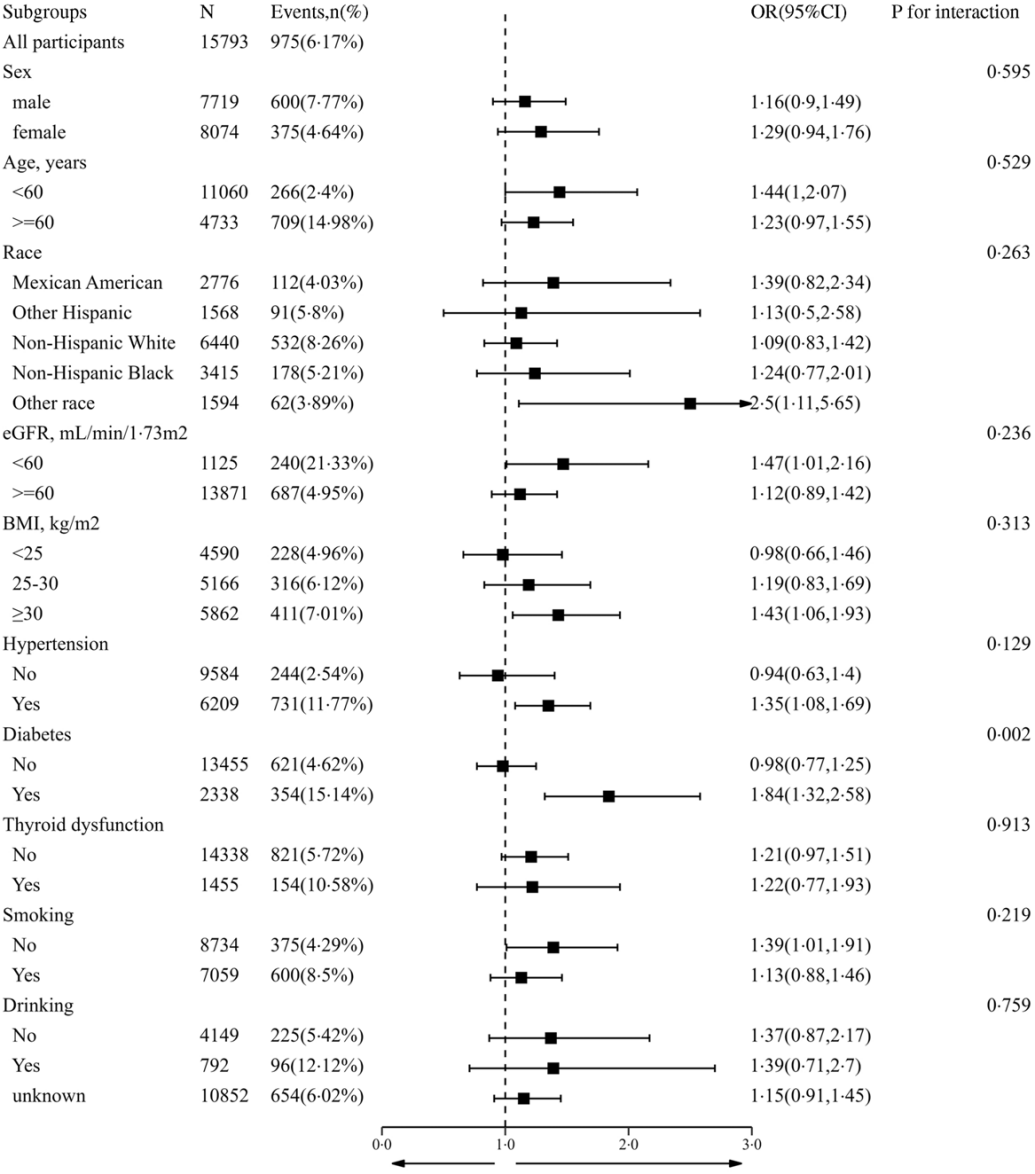
Stratifying analyses by potential modifiers of the association between urinary iodine concentration and coronary artery disease. Each subgroup analysis adjusted for sex, age, race, education levels, marital status, poverty income ratio, BMI, waist circumference, hypertension, thyroid dysfunction, diabetes, smokers, drinkers, fasting plasma glucose, total cholesterol, TAG, uric acid, serum creatinine, estimated glomerular filtration rate, HbA1c and HDL-cholesterol except for the stratifying variable.

**Fig. 3.**
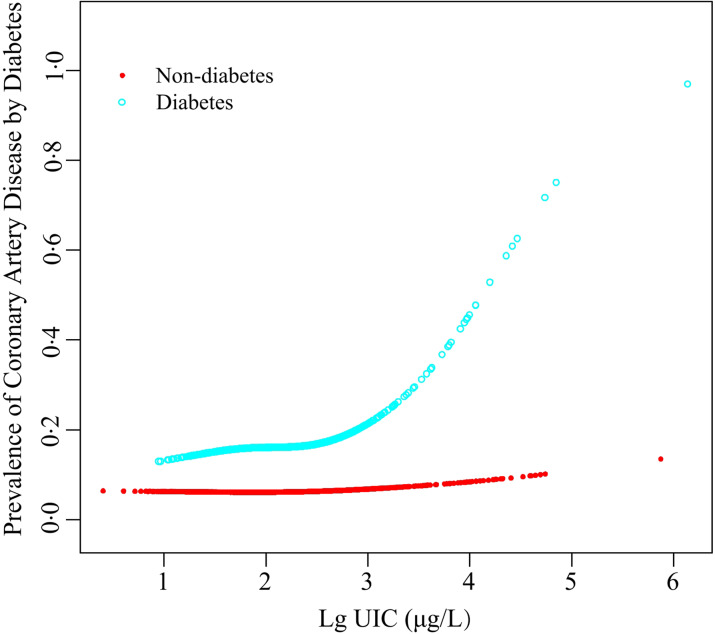
The association between urinary iodine concentration and the prevalence of coronary artery disease is stratified by diabetes. Adjustment factors included sex, age, race, education levels, marital status, poverty income ratio, BMI, waist circumference, hypertension, thyroid dysfunction, smokers, drinkers, fasting plasma glucose, total cholesterol, TAG, uric acid, serum creatinine, estimated glomerular filtration rate, HbA1c and HDL-cholesterol.

The logistic regression analysis results are given in Table 2. In general, UIC was significantly positively associated with CAD in all participants, regardless of whether adjusted for confounding factors. Within the unadjusted model, a positive trend was observed for UIC and CAD (per natural log [UIC] increment: OR, 2·02; 95 % CI 1·74, 2·35). After adjustment for the existence of confounding elements, the trend remained in model 2 (OR, 1·35; 95 % CI 1·15, 1·58) and model 3 (OR, 1·22; 95 % CI 1·00, 1·49). Consistently, the fitted smoothing curve (Fig. 1) showed a nonlinear positive correlation and an apparent threshold effect for UIC and CAD, with the inflection point calculated by the recursive algorithm as 2·65 μg/l (Table 3). In Lg UIC < 2·65 μg/l, UIC had little to no relation to CAD (OR 0·89; 95 % CI 0·68, 1·16). In contrast, for participants with Lg UIC ≥ 2·65 μg/l, the per natural Lg [UI] increment was as follows: OR 2·29; 95 % CI 1·53, 3·43. When converting Lg UIC to a binary variable, the adjusted OR for participants with higher levels of UIC compared with lower levels of UIC (LG UI < 2·65 μg/l) was 1·24 (95 % CI 0·97, 1·59) (Table 2). Table 2 also shows the difference in the relationships for UIC and CAD between participants with and without diabetes. For each unit increase in Lg UIC in diabetic patients, the incidence of CAD increased 1·84-fold (OR 1·84, 95 % CI 1·32, 2·58). However, in non-diabetic patients, UIC did not correlate with CAD (OR 0·98; 95 % CI 0·77, 1·25). There was an interaction between diabetes and UIC (*P*_for interaction_ = 0·014) (Fig. 2). The results of the weighted multiple regression also showed that UIC remained significantly and positively associated with CAD in the diabetic population (OR 1·93, 95 % CI 1·15, 3·24), but not in the non-diabetic population (OR 0·84, 95 % CI 0·59, 1·22). Although this relationship may also exist in the total population, there was insufficient statistical evidence to support it (OR 1·11, 95 % CI 0·79, 1·55) (online Supplementary Table S6). The above findings were also consistent with the results of the stratified curve fit (Fig. 3).

**Table 2.** Relative odds of CAD according to UIC in different models among American adults (Odds rations and 95 % confidence intervals)

Lg UIC, μg/l	Coronary artery disease								
	Model 1			Model 2			Model 3		
	*n* 15 793			*n* 15 793			*n* 11 996		
All participants	OR	95 % CI	*P*	OR	95 % CI	*P*	OR	95 % CI	*P*
Lg UIC (continuous)	2·02	1·74, 2·35	< 0·001	1·35	1·15, 1·58	< 0·001	1·22	1·00, 1·49	0·048
Lg UIC < 2·65	Reference			Reference			Reference		
Lg UIC >= 2·65	1·95	1·61, 2·36	< 0·001	1·41	1·15, 1·73	< 0·001	1·24	0·97, 1·59	0·0829
Diabetes	*n* 2338			*n* 2338			*n* 1664		
Lg UIC (continuous)	2·18	1·68, 2·82	< 0·001	1·70	1·30, 2·23	< 0·001	1·84	1·32, 2·58	0·0004
Lg UIC < 2·65	Reference			Reference			Reference		
Lg UIC >= 2·65	2·22	1·60, 3·08	< 0·001	1·74	1·23, 2·46	0·002	1·76	1·15, 2·69	0·010
Non-diabetes	*n* 13 455			*n* 13 455			*n* 10 332		
Lg UIC (continuous)	1·94	1·61, 2·34	< 0·001	1·19	0·97, 1·45	0·092	0·98	0·77, 1·25	0·8747
Lg UIC < 2·65	Reference			Reference			Reference		
Lg UIC >= 2·65	1·83	1·45, 2·32	< 0·001	1·27	0·99, 1·64	0·060	1·05	0·77, 1·42	0·772

UIC, urinary iodine concentration; Lg UIC, value was log10-transformed. Model 1 adjusts for none; model 2 adjusts for sex, age and race; model 3 adjusts for sex, age, race, education levels, marital status, poverty income ratio, BMI, waist circumference, hypertension, smokers, drinkers, thyroid dysfunction, fasting plasma glucose, total cholesterol, TAG, uric acid, serum creatinine, estimated glomerular filtration rate, HbA1c and HDL-cholesterol.

**Table 3.** Threshold effect analysis of UIC on CAD in US adults (95 % confidence intervals)

Coronary artery disease (*n* 15 793)	Adjusted *β*	95 % CI	*P*
All participants			
Fitting by the standard linear model	1·22	1·00, 1·49	0·048
Fitting by the two-piece-wise linear mode			
Inflection point	2·65		
Lg UIC < 2·65 (μg/l)	0·89	0·68, 1·16	0·392
Lg UIC > 2·65 (μg/l)	2·29	1·53, 3·43	< 0·001
Log-likelihood ratio			< 0·001

UIC, urinary iodine concentration; CAD, coronary artery disease; Lg UIC, value was log10-transformed; adjusted for sex, age, race, education levels, marital status, poverty income ratio, BMI, waist circumference, hypertension, smokers, drinkers, thyroid dysfunction, fasting plasma glucose, total cholesterol, TAG, uric acid, serum creatinine, estimated glomerular filtration rate, HbA1c and HDL-cholesterol.

### Sensitivity analysis

In the interaction and stratification analyses, apart from diabetes, no other confounding elements had a meaningful effect on the association between UIC and CAD, including age, sex, race, BMI, hypertension, thyroid dysfunction, smoking status and drinking status (*P*_for all interactions_ > 0·05) (Fig. 2).

The LDL-cholesterol data for this study were largely missing, and we divided them into two groups by whether they were missing or not to see the distribution of the other variables. We found that the distribution of important demographic characteristics such as age, sex, race, educational attainment and poverty income ratio was generally similar, which could be considered randomly missing (online Supplementary Table S7). For small amounts of missing variables, we used multiple imputations. All variables with missing data have the same distribution in the imputed dataset and the observed complete case data (online Supplementary Table S8). The results from the regression analysis and threshold effects analysis that used only participants with complete data were similar to those conducted on the multiple imputed datasets given here (online Supplementary Table S9–S11).

## Discussion

Findings from this study indicated a significant positive trend between UIC and CAD, an effect that was evident after careful adjustment and in most of the subgroups considered. Further, the generalised additive model and fitted smoothing curves visualised a nonlinear relationship between UIC and CAD. There is a clear threshold effect between them with an inflection point at 2·65 μg/l. In addition, we found that diabetes might modify the association between UIC and CAD in the stratification and interaction analyses. The results showed a significant positive correlation between UIC and CAD in diabetic patients, but not in non-diabetic patients. The above findings were also consistent in the sensitivity analysis but are somewhat different in the weighted regression analysis. The weighted regression analysis showed that although a positive association between UIC and CAD still existed in diabetic patients, the relationship was not significant in the total population and there was insufficient statistical evidence to support whether the association was real, and therefore a larger sample size or more reliable data would be needed to test the relationship in the future.

Iodine is a necessary trace element in the body. Disturbance in iodine status is a major pathogenic factor in thyroid disorders and has been associated with various diseases, such as high BP, disorders of lipid metabolism and insulin resistance^(2,5,11–14)^. Iodine deficiency has been a major public health issue worldwide for several decades. Over two billion individuals worldwide remain at risk of iodine deficiency^(22)^. Therefore, in almost all countries, iodine deficiency has been controlled by adding iodine to salt. Although progress has been made in combating iodine deficiency, with the increasing popularity of iodised salt programmes, a number of people are beginning to have excessive iodine intake^(23)^. The UIC directly reflects dietary iodine intake, as over 90 % of dietary iodine is excreted in the urine. Therefore, UIC is recommended for assessing the iodine status of the body^(15,24)^. An increased UIC was linked to a higher incidence of CAD in our representative US population. Given this correlation, UIC might be a biomarker for a potential predictor of CAD. Furthermore, UIC could be a screening tool for CAD and provide guidance for treatment to avoid overcorrection in iodine-deficient patients.

To date, there have been limited and controversial studies on the association between UIC and the incidence of CAD. In a previous study, Hasanen *et al.* found the highest association with CVD to be iodine status (*r* = −0·83), in which the maximum iodine concentration has been linked to the minimum incidence of CVD^(25)^. Conversely, the incidence of all-cause mortality was higher in patients with excess iodine exposure than in those with moderate iodine exposure in a prospective design (hazard ratio 1·19; 95 % CI 1·04, 1·37)^(26)^. In addition, studies have shown a neutral correlation between iodine status and CAD^(27)^. Differences in study design, sample size, ethnic distribution and control of confounding factors may explain the controversial results among these studies.

In this study, we enrolled a representative US population, and the sample size was large while controlling for common cardiovascular risk factors that included thyroid function. We found several critical new insights. First, we identified a J-shaped correlation between UIC and CAD. We deduce that this could be explained by the fact that iodine is beneficial to humans at appropriate levels. Nevertheless, this benefit disappears or even becomes harmful when iodine is excessive. In numerous animal and human studies, iodine has been linked to several pathogenic mechanisms of CAD: (i) Iodine is known to be an essential micronutrient in the synthesis of thyroid hormone, and thyroid hormone is a necessary regulator of cardiovascular activity that acts on cardiomyocytes, endothelial cells and vascular smooth muscle^(28)^. However, both iodine deficiency and iodine excess could result in thyroid dysfunction^(2,3,5)^, and thyroid dysfunction could induce cardiac and endothelial dysfunction, thereby increasing the risk of CVD^(29)^. (ii) Iodine has both antioxidant and oxidative effects, depending on its concentration, chemical type and oxidative state in the body^(30)^. (iii) Animal studies from as early as the 1930s demonstrated that iodine compounds could prevent the formation of atherosclerosis^(31)^. However, studies have shown that excess iodine can affect BP, blood sugar and lipid metabolism ^(10–14)^, which are critical factors in atherosclerosis.

Second, we noted that in the multivariable logistic regression, generalised additive model and fitted smoothing curves, an interaction might exist between the UIC and diabetes. Diabetic patients had a significant positive correlation between UIC and CAD, while non-diabetic patients did not correlate. Two possible biological mechanisms might underpin this phenomenon. The first interpretation was the direct influence of iodine on insulin resistance and glucose metabolism. Although very little biological research supports this theory, studies have indicated that excess iodine can affect glucose metabolism and increase the risk of diabetes^(32,33)^. A similar view was expressed in another study, which showed that excess iodine would reduce the survival of islet *β*-cells and insulin secretion in mice^(34)^. A further interpretation is that such interaction is facilitated by undiagnosed thyroid dysfunction. According to a previous survey, the estimated mean incidence of undiagnosed hypothyroidism or hyperthyroidism in Europe was 4·94 % and 1·72 %, respectively, while approximately 50 % of hypo- or hyperthyroidism remains undiagnosed^(35)^. Excessive iodine is known to cause thyroid dysfunction, particularly hyperthyroidism, associated with insulin resistance and altered glucose metabolism^(36)^. The above interpretations are inferences based on the results of the current study and need to be supported by large, well-designed prospective studies in the future.

The above findings have important clinical implications for guiding iodine intake and nutritional management of diabetic patients. Firstly, given that there is a J-shaped positive correlation between UIC and the prevalence of CAD with an inflection point at 2·65 μg/l (i.e. 447 μg/l without Lg-transform), which means that the prevalence of CAD increases with increasing UIC when the value is greater than 2·65 μg/l. Therefore, iodine intake should be reduced to decrease the risk of CAD when the UIC exceeds this threshold, particularly in people at high risk for CAD. Secondly, diabetics are at high risk for CAD, and our findings show that excessive UIC significantly increase the incidence of CAD in diabetics. Therefore, when developing a nutritional treatment plan for diabetic patients, appropriate iodine intake needs to be determined on a case-by-case basis to reduce the risk of CAD. In conclusion, nutrition managers and healthcare providers can develop reasonable nutrition programmes based on individual circumstances to avoid health problems caused by iodine deficiency or overdose.

It is important to note that some disease states (e.g. thyroid dysfunction, renal insufficiency, intestinal disorders, etc.)^(37,38)^ and the use of related medications (e.g. thiourea, amiodarone, etc.)^(39,40)^ could affect the absorption, utilisation and metabolism of iodine. In this study, we used thyroid dysfunction and renal functional status for subgroup analysis and interaction analysis and there were consistent findings in all subgroups, so the results of this study apply to the above population. However, we did not collect other data that may affect iodine status, so if extrapolation to specific populations is required caution needs to be exercised in interpretation.

We used data from a representative US population, with a large sample size, and used weighted regression analysis to cope with the complex multi-stage probability sampling of the NHANES data, which allowed our findings to be strongly correlated with the whole US population. Nevertheless, we must also acknowledge important limitations in this research. First, although our best efforts have been made to adjust for confounders, confounding by unknown or unmeasurable factors cannot be completely ruled out. In addition, to minimise the effect of confounding factors on the results, we adjusted for many biological risk factors, including potential mediators (e.g. thyroid function), which may introduce over-adjustment problems. Therefore, it is recommended that in future studies, the relationship between iodine levels and CAD should be investigated by controlling mediating factors reasonably and using simpler models, in order to avoid over-adjustment. Meanwhile, underlying mechanistic research and prospective studies with large samples are also necessary. Second, we, unfortunately, missed a lot of LDL-cholesterol data. However, the fact that almost all-important demographic characteristics were similar in individuals with and without LDL-cholesterol data, and therefore considering LDL-cholesterol to be missing at random, has no impact on our findings. In addition, the relationship between UIC and CAD was consistent in a carefully adjusted subgroup analysis. Third, in this study, only a single-point urine test was performed to estimate iodine status. Repeated sampling may be needed to overcome the daily variability of individuals, but this process would become very complex and expensive. Fourth, self-reporting by participants might be subject to bias. However, questionnaires are an essential part of national health and nutrition surveys, and many studies are published based on questionnaire data. Fifth, the results extrapolated to other countries need to be interpreted with caution because of the differences between countries.

### Conclusion

In conclusion, we found a J-shaped association between UIC and CAD in the US adult population, with an inflection point of 2·65. Meanwhile, this effect was found to be potentially modifiable by diabetes. Future large studies using a longitudinal design with continuous measurements of UIC could further test our main research hypothesis that excess iodine is associated with an increased risk of various risk factors for CHD (e.g., dyslipidaemia, abnormal glucose metabolism, oxidative stress, etc.).

## Supplementary material

For supplementary material accompanying this paper visit https://doi.org/10.1017/S0007114523001277. click here to view supplementary material
