# Supplementary material for: Association study of urinary iodine concentrations and coronary artery disease among adults in the USA: National Health and Nutrition Examination Survey 2003–2018
Source: Br J Nutr. 2023 Jul 10;130(12):2114–22. doi: 10.1017/S0007114523001277 (PMC10657749; doi:10.1017/S0007114523001277)
Supplement: Supplementary file 1 [file S0007114523001277sup.zip › S0007114523001277sup003.docx]

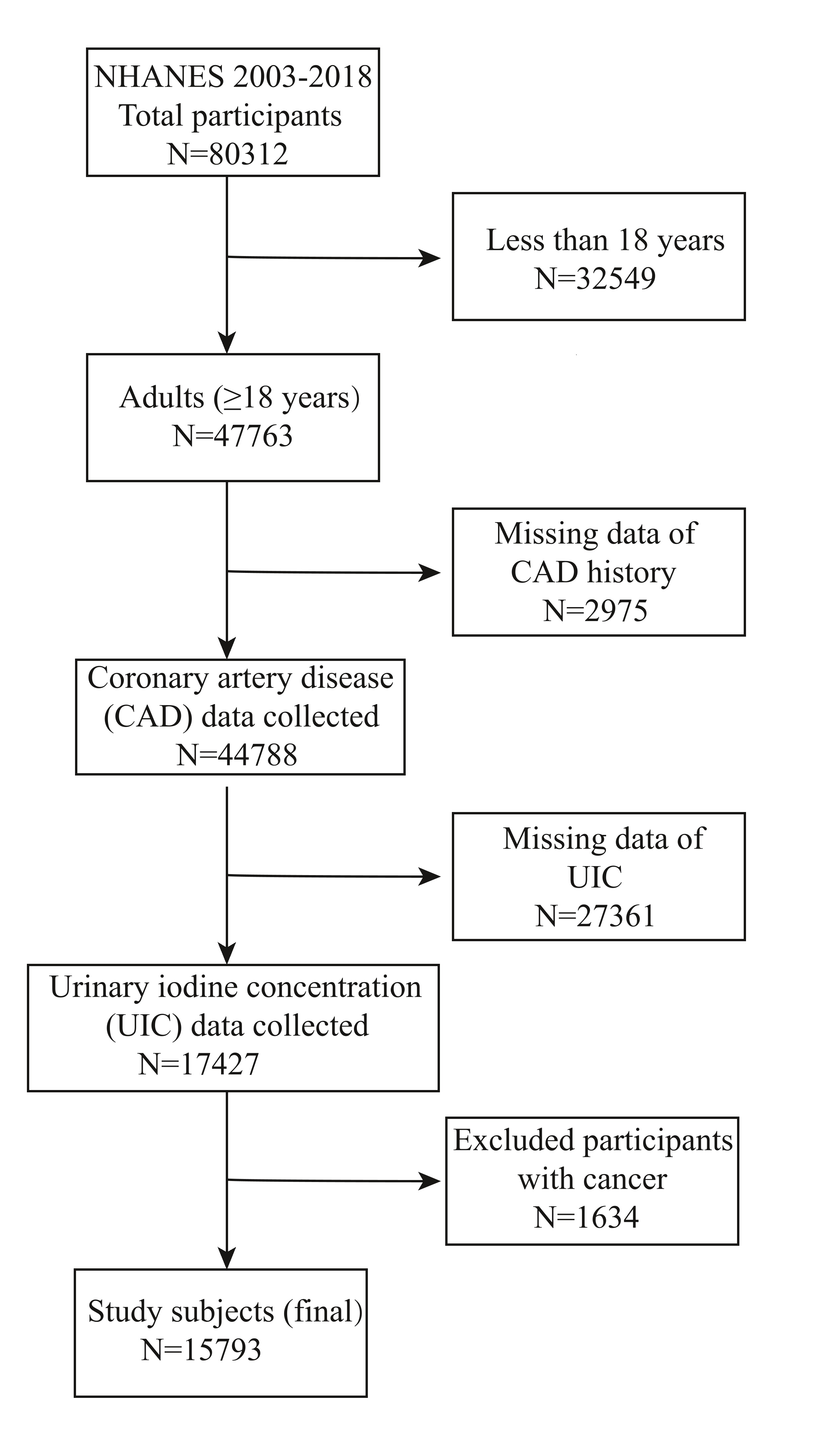


**Figure S1.** Flow diagram of participants’ inclusion


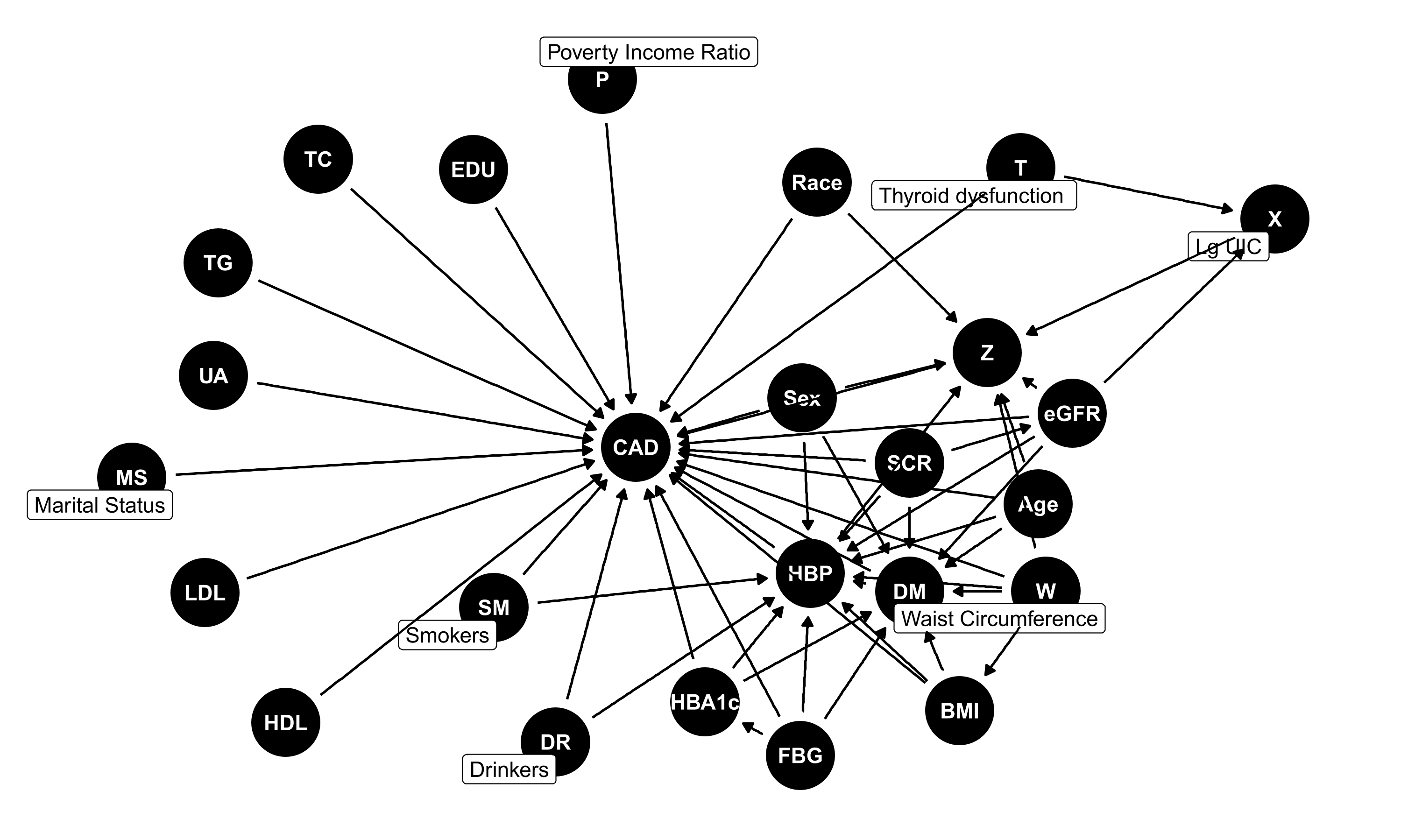


**Figure S2.** Directed acyclic graph for the hypothesized causal relationships.

Abbreviations: X urinary iodine concentration; CAD coronary artery disease; Z thyroid hormone, Oxidative stress or Metabolic disorders; DM diabetes; HBP hypertension; EDU Education level; BMI body mass index; FPG fasting plasma glucose; HbA1c hemoglobin A1c; TC total cholesterol; HDL high-density lipoprotein cholesterol; LDL low-density lipoprotein cholesterol; UA uric acid; SCR Serum creatinine; eGFR estimated glomerular filtration rate.
