# Supplementary material for: Association study of urinary iodine concentrations and coronary artery disease among adults in the USA: National Health and Nutrition Examination Survey 2003–2018
Source: Br J Nutr. 2023 Jul 10;130(12):2114–22. doi: 10.1017/S0007114523001277 (PMC10657749; doi:10.1017/S0007114523001277)
Supplement: Supplementary file 1 [file S0007114523001277sup.zip › S0007114523001277sup002.docx]

Code:

setgam<-function(fml,yi,wdtmp) {

  if (ydist[yi]=="") ydist[yi]<-"gaussian"

  if (ydist[yi]=="exact") ydist[yi]<-"binomial"

  if (ydist[yi]=="breslow") ydist[yi]<-"binomial"

  if (ydist[yi]=="gaussian") mdl<-try(gam(formula(fml),weights=wdtmp$weights,data=wdtmp, family=gaussian(link="identity")))

  if (ydist[yi]=="binomial") mdl<-try(gam(formula(fml),weights=wdtmp$weights,data=wdtmp, family=binomial(link="logit")))

  if (ydist[yi]=="poisson") mdl<-try(gam(formula(fml),weights=wdtmp$weights,data=wdtmp, family=poisson(link="log")))

  if (ydist[yi]=="gamma") mdl<-try(gam(formula(fml),weights=wdtmp$weights,data=wdtmp, family=Gamma(link="inverse")))

  if (ydist[yi]=="negbin") mdl<-try(gam(formula(fml),weights=wdtmp$weights,data=wdtmp, family=negbin(c(1,10), link="log")))

  return(mdl)

}

setgee<-function(fml,yi, wdtmp) {

  if (ydist[yi]=="") ydist[yi]<-"gaussian"

  if (ydist[yi]=="exact") ydist[yi]<-"binomial"

  if (ydist[yi]=="breslow") ydist[yi]<-"binomial"

  if (ydist[yi]=="gaussian") md<-try(geeglm(formula(fml),id=wdtmp[,subjvname],corstr=gee.TYPE,family="gaussian",weights=wdtmp$weights,data=wdtmp))

  if (ydist[yi]=="binomial") md<-try(geeglm(formula(fml),id=wdtmp[,subjvname],corstr=gee.TYPE,family="binomial",weights=wdtmp$weights,data=wdtmp))

  if (ydist[yi]=="poisson") md<-try(geeglm(formula(fml),id=wdtmp[,subjvname],corstr=gee.TYPE,family="poisson",weights=wdtmp$weights,data=wdtmp))

  if (ydist[yi]=="gamma") md<-try(geeglm(formula(fml),id=wdtmp[,subjvname],corstr=gee.TYPE,family="Gamma",weights=wdtmp$weights,data=wdtmp))

  if (ydist[yi]=="negbin") md<-try(geeglm.nb(formula(fml),id=wdtmp[,subjvname],corstr=gee.TYPE,weights=wdtmp$weights,data=wdtmp))

  return(md)

}

setglm<-function(fml,yi, wdtmp) {

  if (ydist[yi]=="") ydist[yi]<-"gaussian"

  if (ydist[yi]=="exact") ydist[yi]<-"binomial"

  if (ydist[yi]=="breslow") ydist[yi]<-"binomial"

  if (ydist[yi]=="gaussian") md<-try(glm(formula(fml),family="gaussian",weights=wdtmp$weights,data=wdtmp))

  if (ydist[yi]=="binomial") md<-try(glm(formula(fml),family="binomial",weights=wdtmp$weights,data=wdtmp))

  if (ydist[yi]=="poisson") md<-try(glm(formula(fml),family="poisson",weights=wdtmp$weights,data=wdtmp))

  if (ydist[yi]=="gamma") md<-try(glm(formula(fml),family="Gamma",weights=wdtmp$weights,data=wdtmp))

  if (ydist[yi]=="negbin") md<-try(glm.nb(formula(fml),weights=wdtmp$weights,data=wdtmp))

  return(md)

}

get.tpval<-function(i,j,fmladj,opt,wdtmp) {

  xTMP <- wdtmp[,xvname[j]]

  tmp.ss<-seq(0.05,0.95,0.05)

  tp<-quantile(xTMP,probs=tmp.ss,na.rm=TRUE)

  tmp.llk<-rep(NA,length(tmp.ss))

  fml<-paste(yvname[i],"~",xvname[j],"+tmp.X",fmladj)

  for (k in (1:length(tmp.ss))) {

    tmp.X<-(xTMP > tp[k])*(xTMP-tp[k]); wdtmp1<-cbind(wdtmp,tmp.X)

    if (opt=="glm" | opt=="gee") tmp.mdl<-setglm(fml, i, wdtmp1);

    if (opt=="gam") tmp.mdl<-setgam(fml, i, wdtmp1);

    tmp.llk[k]<-logLik(tmp.mdl)

    rm(wdtmp1, tmp.X)

  }

  tp1<-tmp.ss[which.max(tmp.llk)]

  tp2.min = tp1 - 0.04

  tp2.max = tp1 + 0.04

  if (tp2.min<0.05) {tp2.min=0.05}

  if (tp2.max>0.95) {tp2.max=0.95}

  tp.pctlrange<-quantile(xTMP,probs=c(tp2.min,tp2.max),na.rm=TRUE)

  tp.range<-unique(xTMP[xTMP>tp.pctlrange[1] & xTMP<tp.pctlrange[2]])

  while (length(tp.range)>5) {

    tmp.pct3<-quantile(tp.range,probs=c(0,0.25,0.5,0.75,1),type=3)

    tmp.llk3<-rep(NA,3)

    for (k in (2:4)) {

      tmp.X<-(xTMP>tmp.pct3[k])*(xTMP-tmp.pct3[k]); wdtmp1<-cbind(wdtmp,tmp.X)

      if (opt=="glm" | opt=="gee") tmp.mdl<-setglm(fml, i, wdtmp1);

      if (opt=="gam") tmp.mdl<-setgam(fml, i, wdtmp1);

      tmp.llk3[k-1]<-logLik(tmp.mdl)

      rm(wdtmp1, tmp.X)

     }

     tmp.min3<-which.max(tmp.llk3)

     tp.range<-tp.range[tp.range>=tmp.pct3[tmp.min3] & tp.range<=tmp.pct3[tmp.min3+2]]

  }

  if (length(tp.range)>0) {

    if (length(tp.range)==1) {tp.val=tp.range[1];} else {

      tmp.llk<-rep(NA,length(tp.range))

      for (k in (1:length(tp.range))) {

         tmp.X<-(xTMP>tp.range[k])*(xTMP-tp.range[k]); wdtmp1<-cbind(wdtmp,tmp.X)

         if (opt=="glm" | opt=="gee") tmp.mdl<-setglm(fml, i, wdtmp1);

         if (opt=="gam") tmp.mdl<-setgam(fml, i, wdtmp1);

         tmp.llk[k]<-logLik(tmp.mdl)

         rm(wdtmp1, tmp.X)

      }

      tp.val<-tp.range[which.max(tmp.llk)]

    }

  } else { tp.val<-tp.pctlrange[1];}

  return(round(tp.val,dec));

}

yvname<-c('Y1', 'Y2');

ydist<-c('gaussian','gaussian');

xvname<-c('X1', 'X2');

fmladj = "+ Z1 + Z2";

for (i in 1:2) {

  for (j in 1:2) {

    tpij <- get.tpval(i, j, fmladj, 'glm', mydata);

  }

}
