# Supplementary material for: Association study of urinary iodine concentrations and coronary artery disease among adults in the USA: National Health and Nutrition Examination Survey 2003–2018
Source: Br J Nutr. 2023 Jul 10;130(12):2114–22. doi: 10.1017/S0007114523001277 (PMC10657749; doi:10.1017/S0007114523001277)
Supplement: Supplementary file 1 [file S0007114523001277sup.zip › S0007114523001277sup001.docx]

**Table S1. Check collinearity (VIF stepwise selection) on original data**

|  | Step 1 | Step 2 |
| --- | --- | --- |
| Lg UIC | 1.1 | 1.1 |
| Age, years | 2.6 | 2.6 |
| Sex | 2.6 | 2.6 |
| Poverty Income Ratio | 1.4 | 1.4 |
| Race | 1.2 | 1.2 |
| Education level | 1.5 | 1.5 |
| Marital Status | 1.2 | 1.2 |
| Weight, kg | 93.9 | NA |
| Height, cm | 22 | 2.8 |
| BMI, kg/m2 | 72 | 8.3 |
| Waist Circumference, cm | 10 | 9.5 |
| HBA1c, % | 3.5 | 3.5 |
| FBG, mmol/L | 2.7 | 2.7 |
| TC, mmol/L | 1.4 | 1.4 |
| Triglyceride, mmol/L | 1.5 | 1.5 |
| HDL-C, mmol/L | 1.6 | 1.6 |
| UA, umol/L | 1.7 | 1.7 |
| SCR, umol/L | 2 | 2 |
| eGFR, mL/min/1.73m^2^ | 2.7 | 2.7 |
| Hypertension | 1.4 | 1.4 |
| Diabetes | 2 | 2 |
| Thyroid dysfunction | 1.1 | 1.1 |
| Smokers | 1.1 | 1.1 |
| Drinkers | 1 | 1 |

First, construct a linear regression equation, explain the variable with all other variables, take the R-squared value of the equation, then calculate VIF= 1/(1-R-squared), if the VIF is greater than or equal to 10 you need to eliminate it. Specifically, the VIF of each variable is calculated first, and if the largest VIF is >= 10 (the default screening criterion), the variable with the largest VIF is removed, and the previous step is repeated until the VIF of all remaining variables is less than the screening criterion

Outcome: coronary artery disease.

Abbreviations: BMI body mass index, FPG fasting plasma glucose, HbA1c hemoglobin A1c, TC total cholesterol, HDL-C high-density lipoprotein cholesterol, LDL-C low-density lipoprotein cholesterol, UA uric acid, SCR Serum creatinine, eGFR estimated glomerular filtration rate, UIC urinary iodine concentration.

Variable removed (VIF>10): Weight.

**Table S2. Associations of covariates with coronary artery disease on original data ^a^**

| Covariates | N | Coronary artery disease | | | |
| --- | --- | --- | --- | --- | --- |
|  |  | OR | 95%CI Low | 95%CI Up | ***P*** |
| Age, years | 15793 | 1.07 | 1.07 | 1.08 | <0.01 |
| Sex |  | | | | |
| Male | 15793 | reference | | | |
| Female |  | 0.58 | 0.51 | 0.66 | <0.01 |
| Poverty Income Ratio | 14425 | 0.85 | 0.81 | 0.88 | <0.01 |
| Height, cm | 15642 | 1.00 | 0.99 | 1.01 | 0.92 |
| BMI, kg/m^2^ | 15618 | 1.02 | 1.01 | 1.03 | <0.01 |
| Waist Circumference, cm | 15110 | 1.02 | 1.02 | 1.03 | <0.01 |
| HBA1c, % | 15190 | 1.34 | 1.29 | 1.40 | <0.01 |
| FBG, mmol/L | 14997 | 1.13 | 1.10 | 1.15 | <0.01 |
| TC, mmol/L | 15039 | 0.70 | 0.65 | 0.74 | <0.01 |
| Triglyceride, mmol/L | 14988 | 1.04 | 1.01 | 1.07 | 0.01 |
| HDL-C, mmol/L | 13587 | 0.53 | 0.44 | 0.64 | <0.01 |
| UA, umol/L | 14992 | 1.00 | 1.00 | 1.01 | <0.01 |
| SCR, umol/L | 14996 | 1.01 | 1.01 | 1.01 | <0.01 |
| eGFR, mL/min/1.73m^2^ | 14996 | 0.97 | 0.97 | 0.97 | <0.01 |
| Race |  | | | | |
| Mexican American | 15793 | reference | | | |
| Other Hispanic |  | 1.47 | 1.10 | 1.95 | 0.01 |
| Non-Hispanic White |  | 2.14 | 1.74 | 2.64 | <0.01 |
| Non-Hispanic Black |  | 1.31 | 1.03 | 1.67 | 0.03 |
| Other race |  | 0.97 | 0.70 | 1.32 | 0.81 |
| Education level |  | | | | |
| <9th grade | 15793 | reference | | | |
| 9–11th grade |  | 0.69 | 0.55 | 0.85 | <0.01 |
| High school |  | 0.71 | 0.58 | 0.86 | <0.01 |
| College |  | 0.51 | 0.41 | 0.62 | <0.01 |
| Graduate or above |  | 0.39 | 0.31 | 0.49 | <0.01 |
| Marital Status |  |  |  |  |  |
| No | 15793 | reference | | | |
| Yes |  | 3.17 | 2.47 | 4.06 | <0.01 |
| Hypertension |  |  |  |  |  |
| No | 15793 | reference | | | |
| Yes |  | 5.11 | 4.40 | 5.93 | <0.01 |
| Diabetes |  |  |  |  |  |
| No | 15793 | reference | | | |
| Yes |  | 3.69 | 3.21 | 4.24 | <0.01 |
| Thyroid dysfunction |  |  |  |  |  |
| No | 15793 | reference | | | |
| Yes |  | 1.95 | 1.63 | 2.34 | <0.01 |
| Smokers |  |  |  |  |  |
| No | 15793 | reference | | | |
| Yes |  | 2.07 | 1.81 | 2.37 | <0.01 |
| Drinkers |  |  |  |  |  |
| No | 15793 | reference | | | |
| Yes |  | 2.41 | 1.87 | 3.10 | <0.01 |
| Unknown |  | 1.12 | 0.96 | 1.31 | 0.16 |

^a^ Values are regression coefficients (95% Confidence Interval) and p-value from univariate regression models and reflect differences in the prevalence of coronary artery disease per unit change of each covariate and for different categories of each covariate as compared to the reference group.

**Table S3. Introduce covariates in the basic model and eliminate covariates in the complete model to observe the change of the regression coefficient of Lg UIC a**

| Covariates | Basic model | Completed model | Choose |
| --- | --- | --- | --- |
| Age, years | 0.4033 * | 0.2840 * | Yes |
| Sex | 0.6906 | 0.2347 * | Yes |
| Poverty Income Ratio | 0.7204 | 0.2147 |  |
| Height, cm | 0.7353 | 0.1983 |  |
| Waist Circumference, cm | 0.6547 * | 0.2050 | Yes |
| HBA1c, % | 0.7453 | 0.1962 |  |
| Triglyceride, mmol/L | 0.7277 | 0.2041 |  |
| HDL-C, mmol/L | 0.6903 | 0.2065 |  |
| UA, umol/L | 0.7238 | 0.2017 |  |
| SCR, umol/L | 0.6940 | 0.1991 |  |
| BMI, kg/m^2^ | 0.7155 | 0.1997 |  |
| FBG, mmol/L | 0.7422 | 0.2023 |  |
| TC, mmol/L | 0.7147 | 0.2199 |  |
| Race | 0.7063 | 0.2244 * | Yes |
| Education level | 0.7049 | 0.2023 |  |
| Marital Status | 0.7174 | 0.2032 |  |
| Hypertension | 0.6235 * | 0.2115 | Yes |
| Diabetes | 0.6852 | 0.2032 |  |
| Thyroid dysfunction | 0.7068 | 0.2098 |  |
| Smokers | 0.7250 | 0.1940 |  |
| Drinkers | 0.7267 | 0.2064 |  |
| eGFR, mL/min/1.73m^2^ | 0.5781 * | 0.2198 | Yes |

^a^ The original regression coefficients for lg UIC were 0.7353 and 0.2027 in the basic and completed models respectively.

^*^Change of more than 10% from the original regression coefficient

**Table S4. Filtered covariates**

| Outcome | exposure | Filtered covariates ^a^ | Filtered covariates ^b^ |
| --- | --- | --- | --- |
| Coronary artery disease | Lg UIC | Age, sex, Waist Circumference, Race, Hypertension, eGFR | Age, sex, Poverty Income Ratio, Waist Circumference, HBA1c, Triglyceride, HDL-C, UA, SCR, BMI, FBG, TC Race, Education level, Marital Status, Hypertension, Diabetes, Thyroid dysfunction Smokers, Drinkers, eGFR |

^a^ Introduction of covariates in the basic model or removal of covariates from the completed model has >10% impact on the regression coefficient of Lg UIC.

^b^ Criterion (a) or P-value of regression coefficient of covariates on Y < 0.1.

**Table S5. Weighted characteristics of the study population**

| Variables | Non-CAD(n=14818) | CAD(n=975) | ***P*** |
| --- | --- | --- | --- |
| Age, years | 44.57 (44.09, 45.04) | 63.41 (62.25 ,64.56) | <0.001 |
| Sex |  |  | <0.001 |
| male | 48.00 (46.93,49.08) | 59.75 (55.04 ,64.29) |  |
| female | 52.00 (50.92,53.07) | 40.25 (35.71 ,44.96) |  |
| Race, % |  |  | <0.001 |
| Mexican American | 9.35 (8.02,10.86) | 4.63 (3.18 ,6.71) |  |
| Other Hispanic | 5.91 (5.08,6.87) | 4.12 (2.99 ,5.65) |  |
| Non-Hispanic White | 64.21 (61.66,66.68) | 76.31 (72.25 ,79.94) |  |
| Non-Hispanic Black | 12.12 (10.83,13.55) | 9.36 (7.45 ,11.70) |  |
| Other race | 8.41 (7.51,9.41) | 5.58 (3.85 ,8.01) |  |
| Marital Status |  |  | <0.001 |
| No | 19.82 (18.59,21.11) | 6.81 (4.58 ,10.02) |  |
| Yes | 80.18 (78.89 ,81.41) | 93.19 (89.98 ,95.42) |  |
| Education level, % |  |  | <0.001 |
| <9th grade | 5.87 (5.31 ,6.49) | 10.22 (8.30 ,12.53) |  |
| 9–11th grade | 10.42 (9.63 ,11.27) | 14.39 (11.56 ,17.77) |  |
| High school | 23.75 (22.66 ,24.87) | 29.07 (24.48 ,34.13) |  |
| College | 31.24 (30.05 ,32.46) | 28.17 (23.83 ,32.96) |  |
| Graduate or above | 28.71 (26.82 ,30.68) | 18.15 (14.04 ,23.15) |  |
| Poverty Income Ratio | 2.94 (2.88 ,3.00) | 2.56 (2.38 ,2.75) | <0.001 |
| Weight, kg | 82.38 (81.76 ,83.01) | 85.37 (83.74 ,86.99) | 0.001 |
| Height, cm | 168.69 (168.44 ,168.95) | 168.06 (167.16 ,168.96) | 0.175 |
| BMI, kg/m^2^ | 28.86 (28.65 ,29.06) | 30.14 (29.63 ,30.64) | <0.001 |
| Waist Circumference, cm | 98.39 (97.86 ,98.92) | 105.53 (104.25 ,106.81) | <0.001 |
| Hypertension |  |  | <0.001 |
| No | 67.62 (66.38 ,68.84) | 31.00 (26.50 ,35.88) |  |
| Yes | 32.38 (31.16 ,33.62) | 69.00 (64.12 ,73.50) |  |
| Diabetes |  |  | <0.001 |
| No | 90.39 (89.71 ,91.02) | 68.06 (63.96 ,71.90) |  |
| Yes | 9.61 (8.98 ,10.29) | 31.94 (28.10 ,36.04) |  |
| Thyroid dysfunction |  |  | <0.001 |
| No | 90.39 (89.66 ,91.07) | 80.88 (77.31 ,84.00) |  |
| Yes | 9.61 (8.93 ,10.34) | 19.12 (16.00 ,22.69) |  |
| Smokers |  |  | <0.001 |
| No | 56.27 (54.80 ,57.74) | 38.33 (33.76 ,43.11) |  |
| Yes | 43.73 (42.26 ,45.20) | 61.67 (56.89 ,66.24) |  |
| Drinkers |  |  | <0.001 |
| No | 36.31 (34.63 ,38.02) | 36.91 (32.06 ,42.04) |  |
| Yes | 6.49 (5.72 ,7.35) | 13.31 (10.35 ,16.97) |  |
| Unknown | 57.21 (55.41 ,58.98) | 49.77 (44.35 ,55.21) |  |
| LDL-C |  |  | 0.595 |
| Know | 55.44 (54.25 ,56.61) | 53.96 (48.69 ,59.14) |  |
| Unknown | 44.56 (43.39 ,45.75) | 46.04 (40.86 ,51.31) |  |
| TC, mmol/L | 5.06 (5.03 ,5.09) | 4.71 (4.59 ,4.82) | <0.001 |
| Triglyceride, mmol/L | 1.71 (1.68 ,1.75) | 1.91 (1.80 ,2.02) | 0.001 |
| HDL-C, mmol/L | 1.38 (1.37 ,1.40) | 1.31 (1.26 ,1.35) | 0.001 |
| FBG, mmol/L | 5.45 (5.41 ,5.48) | 6.35 (6.15 ,6.54) | <0.001 |
| HBA1c, % | 5.56 (5.54 ,5.58) | 6.13 (6.02 ,6.23) | <0.001 |
| UA, umol/L | 319.39 (317.34 ,321.44) | 348.26 (339.65 ,356.87) | <0.001 |
| SCR, umol/L | 77.10 (76.59 ,77.60) | 89.91 (87.07 ,92.74) | <0.001 |
| eGFR, mL/min/1.73m^2^ | 95.25 (94.56 ,95.94) | 79.36 (77.07 ,81.66) | <0.001 |
| UIC, μg/L | 240.26 (193.85 ,286.67) | 1255.73 (223.27 ,2288.20) | 0.056 |
| Lg UIC ^a^, μg/L | 2.12 (2.11 ,2.13) | 2.23 (2.18 ,2.29) | <0.001 |

Abbreviations: BMI body mass index, FPG fasting plasma glucose, HbA1c hemoglobin A1c, TC total cholesterol, HDL-C high-density lipoprotein cholesterol, LDL-C low-density lipoprotein cholesterol, UA uric acid, SCR Serum creatinine, eGFR estimated glomerular filtration rate, UIC urinary iodine concentration.

^a^ UIC value was log10-transformed.

For continuous variables: survey-weighted mean (95% CI), P-value was by survey-weighted linear regression (svyglm).

For categorical variables: survey-weighted percentage (95% CI), P-value was by survey-weighted Chi-square test (svytable).

**Table S6. Weighted relative odds of CAD according to UIC in different models among American adults**

| Lg UIC, μg/L | Coronary artery disease OR (95% CI), P value | | |
| --- | --- | --- | --- |
|  | Model1 | Model2 | Model3 |
| All participants (N=15793) |  | | |
| Lg UIC | 1.82 (1.38, 2.40) <0.001 | 1.16 (0.84, 1.62) 0.359 | 1.11 (0.79, 1.55) 0.572 |
| Diabetes (N=2338) |  | | |
| Lg UIC | 2.50 (1.63, 3.85) <0.001 | 1.70 (1.30, 2.23) <0.001 | 1.93 (1.15, 3.24) 0.014 |
| Non-Diabetes |  | | |
| Lg UIC | 1.57 (1.12, 2.20) 0.01 | 0.92 (0.64, 1.33) 0.67 | 0.84 (0.59, 1.22) 0.374 |

Abbreviations: UIC urinary iodine concentration, Lg UIC value was log10-transformed. Model 1 adjusts for none; Model 2 adjusts for sex, age, and race; Model 3 adjusts for sex, age, race, education levels, marital status, poverty income ratio, body mass index, waist circumference, hypertension, smokers, drinkers, thyroid dysfunction, fasting plasma glucose, total cholesterol, triglyceride, uric acid, Serum creatinine, estimated glomerular filtration rate, hemoglobin A1c, high-density lipoprotein cholesterol.

**Table S7. Sensitivity comparative analysis between participants with vs. without known LDL-C data**

| Variables ^a^ | LDL-C(unknown)  N=8748 | LDL-C(known)  N=7045 | ***P***-Value |
| --- | --- | --- | --- |
| Age, years | 47.65 ± 17.35 | 48.55 ± 17.51 | 0.001 |
| Male, % | 4265 (48.75%) | 3454 (49.03%) | 0.732 |
| Race, % |  |  | 0.016 |
| Mexican American | 1517 (17.34%) | 1259(17.87%) |  |
| Other Hispanic | 834(9.57%) | 731(10.38%) |  |
| Non-Hispanic White | 3522(40.26%) | 2918(41.42%) |  |
| Non-Hispanic Black | 1961(22.42%) | 1454(20.64%) |  |
| Other race | 911(10.41%) | 683(9.69%) |  |
| Marital Status |  |  | 0.014 |
| No | 1692(19.34%) | 1255(17.81%) |  |
| Yes | 7056(80.66%) | 5790(82.19%) |  |
| Education level, % |  |  | 0.313 |
| <9th grade | 1053(12.04%) | 853(12.11%) |  |
| 9–11th grade | 1289(14.73%) | 1083(15.37%) |  |
| High school | 2083(23.81%) | 1620(23.00%) |  |
| College | 2504(28.62%) | 1958(27.79%) |  |
| Graduate or above | 1819(20.79%) | 1531(21.73%) |  |
| Poverty Income Ratio | 2.51 ± 1.56 | 2.48 ± 1.54 | 0.256 |
| Weight, kg | 81.66 ± 21.53 | 81.21 ± 20.93 | 0.188 |
| Height, cm | 167.00 ± 10.15 | 167.36 ± 10.03 | 0.025 |
| BMI, kg/m^2^ | 29.19 ± 6.91 | 28.91 ± 6.67 | 0.009 |
| Waist Circumference, cm | 99.06 ± 16.05 | 98.71 ± 15.58 | 0.170 |
| Hypertension |  |  | 0.825 |
| No | 5302 (60.61%) | 4282(60.78%) |  |
| Yes | 3446(39.39%) | 2763(39.22%) |  |
| Diabetes |  |  | 0.065 |
| No | 7412 (84.73%) | 6043 (85.78%) |  |
| Yes | 1336 (15.27%) | 1002 (14.22%) |  |
| Thyroid dysfunction |  |  | 0.109 |
| No | 7971 (91.12%) | 6367 (90.38%) |  |
| Yes | 777 (8.88%) | 678 (9.62%) |  |
| Smokers |  |  | 0.317 |
| No | 4869 (55.66%) | 3865 (54.86%) |  |
| Yes | 3879 (44.34%) | 3180 (45.14%) |  |
| Drinkers |  |  | <0.001 |
| No | 2299 (26.28%) | 1850 (26.26%) |  |
| Yes | 428 (4.89%) | 364 (5.17%) |  |
| Unknow | 6021 (68.83%) | 4831 (68.57%) |  |
| FBG, mmol/L | 5.69 ± 2.25 | 5.65 ± 1.92 | 0.258 |
| HBA1c, % | 5.74 ± 1.04 | 5.72 ± 1.05 | 0.206 |
| TC, mmol/L | 5.07 ± 1.06 | 5.01 ± 1.06 | <0.001 |
| Triglyceride, mmol/L | 2.09 ± 1.96 | 1.37 ± 0.77 | <0.001 |
| HDL-C, mmol/L | 1.34 ± 0.38 | 1.41 ± 0.39 | <0.001 |
| UA, umol/L | 321.39 ± 82.81 | 326.38 ± 83.70 | <0.001 |
| SCR, umol/L | 79.04 ± 31.10 | 77.91 ± 31.83 | <0.001 |
| eGFR, mL/min/1.73m^2^ | 93.30 ± 26.84 | 95.18 ± 26.82 | <0.001 |
| UIC ^b^, μg/L | 2.16 ± 0.42 | 2.13 ± 0.38 | <0.001 |

BMI body mass index, FPG fasting plasma glucose, HbA1c hemoglobin A1c, TC total cholesterol, HDL-C high-density lipoprotein cholesterol, LDL-C low-density lipoprotein cholesterol, UA uric acid, SCR Serum creatinine, eGFR estimated glomerular filtration rate, UIC urinary iodine concentration.

^a^ Data are presented as number (%) or mean ± standard deviation

^b^ UIC value was log10-transformed

**Table S8. Distribution of variables with missing data comparing the raw data with the data by multiple imputation**

| Variables | Raw data | MI.1 | MI.2 | MI.3 | MI.4 | MI.5 | P-value |
| --- | --- | --- | --- | --- | --- | --- | --- |
| Poverty Income Ratio | 2.50 ± 1.62 | 2.48 ± 1.61 | 2.48 ± 1.61 | 2.48 ± 1.61 | 2.48 ± 1.62 | 2.48 ± 1.62 | 0.880 |
| Weight, kg | 81.46 ± 21.37 | 81.44 ± 21.36 | 81.43 ± 21.37 | 81.45 ± 21.38 | 81.43 ± 21.35 | 81.46 ± 21.37 | 1.000 |
| Height, cm | 167.16 ± 10.15 | 167.14 ± 10.14 | 167.14 ± 10.16 | 167.14 ± 10.15 | 167.14 ± 10.15 | 167.14 ± 10.15 | 1.000 |
| BMI, kg/m^2^ | 29.07 ± 6.85 | 29.07 ± 6.85 | 29.07 ± 6.85 | 29.08 ± 6.85 | 29.08 ± 6.85 | 29.08 ± 6.85 | 1.000 |
| Waist Circumference, cm | 98.90 ± 16.20 | 99.01 ± 16.32 | 99.02 ± 16.34 | 99.01 ± 16.34 | 99.00 ± 16.34 | 99.01 ± 16.34 | 0.990 |
| FBG, mmol/L | 5.67 ± 2.17 | 5.68 ± 2.14 | 5.67 ± 2.14 | 5.67 ± 2.14 | 5.67 ± 2.14 | 5.68 ± 2.14 | 0.999 |
| HBA1c, % | 5.73 ± 1.07 | 5.73 ± 1.06 | 5.73 ± 1.06 | 5.73 ± 1.06 | 5.73 ± 1.06 | 5.73 ± 1.06 | 0.994 |
| TC, mmol/L | 5.05 ± 1.09 | 5.05 ± 1.09 | 5.05 ± 1.09 | 5.05 ± 1.09 | 5.06 ± 1.09 | 5.07 ± 1.09 | 0.723 |
| Triglyceride, mmol/L | 1.77 ± 1.63 | 1.77 ± 1.62 | 1.78 ± 1.62 | 1.78 ± 1.62 | 1.78 ± 1.62 | 1.78 ± 1.62 | 0.975 |
| HDL-C, mmol/L | 1.37 ± 0.42 | 1.37 ± 0.42 | 1.37 ± 0.42 | 1.37 ± 0.42 | 1.37 ± 0.42 | 1.37 ± 0.42 | 1.000 |
| UA, umol/L | 323.65 ± 85.44 | 324.01 ± 85.74 | 324.06 ± 85.37 | 324.30 ± 85.77 | 324.26 ± 85.95 | 324.10 ± 85.62 | 0.990 |
| SCR, umol/L | 78.54 ± 32.25 | 78.62 ± 31.82 | 78.63 ± 31.85 | 78.71 ± 31.86 | 78.81 ± 31.92 | 78.72 ± 31.88 | 0.983 |
| eGFR, mL/min/1.73m^2^ | 94.55 ± 27.29 | 94.34 ± 27.35 | 94.38 ± 27.48 | 94.24 ± 27.36 | 94.14 ± 27.41 | 94.27 ± 27.46 | 0.855 |

**Table S9.** **Relative odds of CAD according to UIC in different model among American adults based on multiple imputed data**

| Lg UIC, μg/L | Coronary artery disease (OR (95% CI)) | | | | | | | | | |
| --- | --- | --- | --- | --- | --- | --- | --- | --- | --- | --- |
|  | Model 1 | | | | | Model 2 | | | | |
|  | MI.data 1 | MI.data 2 | MI.data 3 | MI.data 4 | MI.data 5 | MI.data 1 | MI.data 2 | MI.data 3 | MI.data 4 | MI.data 5 |
| **All participants (N=15793)** |  |  |  |  |  |  |  |  |  |  |
| Lg UIC | 2.02 (1.74, 2.35) | 2.02 (1.74, 2.35) | 2.02 (1.74, 2.35) | 2.02 (1.74, 2.35) | 2.02 (1.74, 2.35) | 1.27 (1.07, 1.50) | 1.26 (1.07, 1.48) | 1.26 (1.07, 1.49) | 1.25 (1.06, 1.48) | 1.26 (1.07, 1.49) |
| Lg UIC < IP ^a^ | Reference | Reference | Reference | Reference | Reference | Reference | Reference | Reference | Reference | Reference |
| Lg UIC>= IP ^a^ | 1.87 (1.56, 2.25) | 1.87 (1.56, 2.25) | 1.87 (1.56, 2.25) | 1.87 (1.56, 2.25) | 1.87 (1.56, 2.25) | 1.26 (1.03, 1.54) | 1.26 (1.03, 1.55) | 1.27 (1.04, 1.56) | 1.26 (1.03, 1.55) | 1.28 (1.04, 1.56) |
| **Diabetes (N=2338)** |  |  |  |  |  |  |  |  |  |  |
| Lg UI (Continuous) | 2.18 (1.68, 2.82) | 2.18 (1.68, 2.82) | 2.18 (1.68, 2.82) | 2.18 (1.68, 2.82) | 2.18 (1.68, 2.82) | 1.70 (1.28, 2.25) | 1.66 (1.25, 2.20) | 1.67 (1.26, 2.22) | 1.66 (1.25, 2.20) | 1.66 (1.25, 2.20) |
| Lg UIC < IP ^a^ | Reference | Reference | Reference | Reference | Reference | Reference | Reference | Reference | Reference | Reference |
| Lg UIC>= IP ^a^ | 2.15 (1.56, 2.96) | 2.15 (1.56, 2.96) | 2.15 (1.56, 2.96) | 2.15 (1.56, 2.96) | 2.15 (1.56, 2.96) | 1.75 (1.24, 2.47) | 1.70 (1.20, 2.41) | 1.73 (1.22, 2.45) | 1.71 (1.21, 2.41) | 1.71 (1.21, 2.41) |
| **Non-Diabetes (N=13455)** |  |  |  |  |  |  |  |  |  |  |
| Lg UI (Continuous) | 1.94 (1.61, 2.34) | 1.94 (1.61, 2.34) | 1.94 (1.61, 2.34) | 1.94 (1.61, 2.34) | 1.94 (1.61, 2.34) | 1.09 (0.89, 1.34) | 1.09 (0.89, 1.34) | 1.09 (0.89, 1.34) | 1.09 (0.89, 1.34) | 1.11 (0.90, 1.36) |
| Lg UIC < IP ^a^ | Reference | Reference | Reference | Reference | Reference | Reference | Reference | Reference | Reference | Reference |
| Lg UIC>= IP ^a^ | 1.75 (1.39, 2.20) | 1.75 (1.39, 2.20) | 1.75 (1.39, 2.20) | 1.75 (1.39, 2.20) | 1.75 (1.39, 2.20) | 1.09 (0.84, 1.40) | 1.09 (0.85, 1.41) | 1.08 (0.84, 1.40) | 1.08 (0.84, 1.40) | 1.10 (0.86, 1.42) |

Abbreviations: MI multiple imputed; IP inflection point; Lg UIC value was log10-transformed. Model 1 adjusts for none; Model 2 adjusts for sex, age, race, education levels, marital status, poverty income ratio, weight, height, body mass index, waist circumference, hypertension, smokers, drinkers, thyroid dysfunction, fasting plasma glucose, total cholesterol, triglyceride, uric acid, Serum creatinine, estimated glomerular filtration rate, hemoglobin A1c, high-density lipoprotein cholesterol. ^a^ IP=2.63 in MI.data 1, 2, 3, and 4, while IP=2.62 in MI.data 5.

**Table S10. Pool estimates from multiple imputed data**

| Data | | | | | | | | | | | | | |
| --- | --- | --- | --- | --- | --- | --- | --- | --- | --- | --- | --- | --- | --- |
| Var^*^ | beta 1 | Se 1 | beta 2 | Se 2 | beta 3 | | Se 3 | | beta 4 | | Se 4 | beta 5 | Se 5 |
| Lg UIC | 0.231112 | 0.084469 | 0.223144 | 0.085146 | 0.231112 | | 0.085146 | | 0.223144 | | 0.085146 | 0.231112 | 0.084469 |
| Pooled estimates | | | | | | | | | | | | | |
| Var. | beta | Se | P value | exp(beta) | | 95%CI low | | 95%CI up | |  |  |  |  |
| Lg UIC | 0.227924 | 0.084988 | 0.007322 | 1.255990 | | 1.063269 | | 1.483644 | |  |  |  |  |

^*^ 1, 2, 3, 4, 5 refer to MI.data1, MI.data2, MI.data3, MI.data4, MI.data5, respectively.

**Table S11. Comparison of the results of threshold effect analysis of UIC on CAD among US adults on imputed data**

|  | MI.data 1 | MI.data 2 | MI.data 3 | MI.data 4 | MI.data 5 |
| --- | --- | --- | --- | --- | --- |
| Coronary artery disease | Adjusted β (95% CI) | | | | |
| All participants |  |  |  |  |  |
| Fitting by the standard linear model | 1.27 (1.07, 1.50) 0.005 | 1.26 (1.07, 1.48) 0.007 | 1.26 (1.07, 1.49) 0.006 | 1.25 (1.06, 1.48) 0.007 | 1.26 (1.07, 1.49) 0.006 |
| Fitting by the two-piecewise linear mode |  |  |  |  |  |
| Inflection point | 2.63 | 2.63 | 2.63 | 2.63 | 2.62 |
| Lg UIC < Inflection point | 0.96 (0.77, 1.20) 0.725 | 0.95 (0.76, 1.20) 0.681 | 0.96 (0.76, 1.20) 0.704 | 0.95 (0.76, 1.19) 0.674 | .95 (0.76, 1.19) 0.673 |
| Lg UIC > Inflection point | 2.18 (1.55, 3.07) <0.001 | 2.15 (1.53, 3.03) <0.001 | 2.15 (1.53, 3.02) <0.001 | 2.13 (1.52, 3.00) <0.001 | 2.15 (1.53, 3.01) <0.001 |
| Log likelihood ratio | <0.001 | <0.001 | <0.001 | <0.001 | <0.001 |

UIC urinary iodine concentration, CAD coronary artery disease, Lg UIC value was log10-transformed; adjust for: gender, age, race, education levels, marital status, poverty income ratio, weight, height, body mass index, waist circumference, hypertension, smokers, drinkers, thyroid dysfunction, fasting plasma glucose, total cholesterol, triglyceride, uric acid, Serum creatinine, estimated glomerular filtration rate, hemoglobin A1c, high-density lipoprotein cholesterol.

**Table S12. Relative odds of CAD according to UIC in different model among American adults based on raw data** **without Lg-transform**

| UIC, mg/L | Coronary artery disease (OR (95% CI) p-value) | | |
| --- | --- | --- | --- |
|  | Model 1 | Model 2 | Model 3 |
| **All participants** | 1.004 (0.999, 1.009) 0.0848 | 1.005 (1.000, 1.010) 0.0467 | 1.004 (0.999, 1.010) 0.1091 |
| **Non-Diabetes** | 1.003 (0.997, 1.008) 0.4069 | 1.004 (0.996, 1.011) 0.3131 | 1.001 (0.987, 1.016) 0.8498 |
| **Diabetes** | 1.105 (1.030, 1.185) 0.0054 | 1.081 (1.016, 1.149) 0.0132 | 1.064 (1.011, 1.120) 0.0175 |

Abbreviations: UIC urinary iodine concentration. Model 1 adjusts for none; Model 2 adjusts for sex, age, and race; Model 3 adjusts for sex, age, race, education levels, marital status, poverty income ratio, weight, height, body mass index, waist circumference, hypertension, smokers, drinkers, thyroid dysfunction, fasting plasma glucose, total cholesterol, triglyceride, uric acid, Serum creatinine, estimated glomerular filtration rate, hemoglobin A1c, high-density lipoprotein cholesterol.
